# Coarse-Grained Simulations of Thermosensitive Polymer Nanocomposites

**DOI:** 10.1021/acs.macromol.5c02656

**Published:** 2026-02-05

**Authors:** María del Mar Ramos-Tejada, Alberto Martín-Molina, Daniel Montesinos, Luis Pérez-Mas, Manuel Quesada-Pérez

**Affiliations:** † Departamento de Física, Escuela Politécnica Superior de Linares, Universidad de Jaén, Linares, Jaén 23700, Spain; ‡ Departamento de Física Aplicada, Universidad de Granada, Campus de Fuentenueva s/n, Granada 18071, Spain; § Instituto Carlos I de Física Teórica y Computacional, Universidad de Granada, Campus de Fuentenueva s/n, Granada 18071, Spain; ∥ 16379 Instituto de Física Interdisciplinar y Sistemas Complejos (IFISC) Campus Universitat de les Illes Balears Carretera de Valldemossa, Km 7,5 Edificio Científico-Técnico, Palma de Mallorca, Islas Baleares 07122, Spain

## Abstract

Nanogels (as well as other polymer networks) can absorb nanoparticles that give them new properties and expand their application possibilities. The resulting hybrid entities constitute a kind of polymer nanocomposites, which have become an emerging area of research. In this work, coarse-grained simulations have been used to study how certain properties of these nanocomposites (size, number of nanoparticles inside, net charge, and surface potential) change with temperature. Four nanocomposites with different values of charge anchored to the polymer network (known as bare charge) were simulated. The degree to which nanocomposites shrink and expel the particles they contain depends strongly on the bare charge, which, in turn, could be related to the pH in pH-sensitive micro- and nanogels. Our results also reveal that nanoparticles are responsible for nanocomposites exhibiting much richer and more complex behavior than nanogels. Furthermore, the strong correlations that nanoparticles experience when the polymer network shrinks should not be ignored in mean-field theories that try to predict how nanocomposites behave.

## Graphical abstract

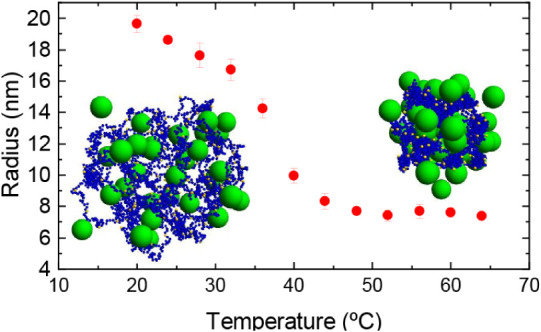

## Introduction

One of the most important properties of nanogels is their ability to absorb ions, drugs, macromolecules, polyelectrolytes and even other organic and inorganic nanoparticles. The systems formed by nanogels and the nanoparticles they absorb are known as hybrids, nanoparticle-nanogel composites or nanocomposites.^1^ The latter will be the name used in this work. These nanomaterials offer a wide range of potential applications in nanomedicine, including not only drug delivery but also imaging and theranostics.^1^

In the past decade, coarse-grained (CG) models (implemented through computer simulation techniques) have contributed to a better understanding of the behavior of nanogels.^2–11^ Previously, these models had also been applied to polyelectrolyte gels,^12–20^ and solute diffusion in networks.^21–25^ Computer simulations (at different scales) are also used in the study of different biotechnological applications of hydrogels, micro- and nanogels.^26,27^ One of the great advantages of coarse-grained simulations is that they allow us to consider aspects that are very difficult to include in other theoretical treatments, such as chain fluctuations, the complex topology of the polymeric network, or discrete charge distributions. Furthermore, coarse-grained models allow simulating larger systems than atomistic simulations.

In a previous work, the absorption of nanoparticles in oppositely charged nanogels was simulated with the help of a coarse-grained model.^28^ These simulations provided valuable information on the properties of these nanocomposites (size, effective charge, electrostatic potential, nanoparticle distribution) and were able to explain previously reported experimental results, such as the reversal in electrophoretic mobility reported for a variety of real systems of different nature.^29–33^

However, it should be mentioned that such simulations were restricted to absorption into swollen nanogels and, therefore, the behavior of the formed nanocomposites was mainly governed by electrostatic forces. One of the main objectives of this work is to extend the previous study to thermo-shrinking nanocomposites. Several authors propose the use of thermo-shrinking nanogels to deliver drugs by heating them.^34–37^ This strategy works with small molecules. For example, Cazares has shown that the fraction of drug released by these nanogels increases with temperature.^37^ However, the question arises as to whether nanoparticles of moderate size can escape from a collapsing polymeric network. This is not a trivial question. As the solute size and polymer volume fraction increase, the role of steric forces may be decisive. The polymer chains that form a gel, micro- or nanogel become an obstacle to the diffusion of nanoparticles contained within it. What is more, the nanoparticles could be trapped in the voids of the network if the polymer volume fraction is high enough.^22^ It would be interesting to find out to what extent nanoparticles encapsulated in a nanogel are expelled when the polymeric network shrinks in response to a change in temperature. In addition, this work aims to shed light on the mechanisms that drive nanoparticles from the innermost region of the nanogel to its surface. It is also interesting to find out how nanoparticles with considerable charge and size (compared to those of ions) alter the properties of the nanogels in which they are absorbed. The coarse-grained model used in this study is essentially the same as the one used in the previous work.^28^ The main difference is that it includes solvent-mediated hydrophobic forces that induce the shrinkage of nanocomposites upon heating. Simulations were performed through Monte Carlo techniques.

The rest of the paper is organized as follows. First, the models and methodology used in the simulations are described. Next, the most relevant results are presented and discussed. Finally, some conclusions are drawn.

## Model and Simulations

### Basic Constituents of the CG Model

According to our coarse-grained model, monomeric units, cross-linkers, nanoparticles, and ions are modeled as spheres (or beads). The diameters of these species can be found in Table 1 and have been used previously.^2^ In the case of ions, these diameters include the hydration shell. The solvent (water) is considered a continuous dielectric medium.

**Table 1:** Parameters of the Model

Parameter	Value
Diameter of monomeric units and cross-linkers	0.65 nm
Diameter of ions	0.70 nm
Diameter of nanoparticles	5.00 nm
ε_WCA_	4.11 × 10^–21^ J
*k*_e_	0.40 N/m
*r*_0_	0.65 nm
*k_h_*	12.1 nm^–1^
*r_h_*	0.90 nm
ε_max_	5.5 × 10^–21^ J
*T*_ε/2_	307.5 K
*k*_ε/2_	0.0667 K^–1^

Four nanogels, referred to as NG1, NG2, NG3 and NG4, have been used in this study. The number that identifies them is also the number of charged monomers per chain. For example, NG3 has three charged monomers per chain. The electric charge of each charged monomeric unit is +*e* (the elementary charge). The polymer network of these nanogels is made up of 100 polyelectrolyte chains. Therefore, the electric charge (expressed in elementary units) anchored to the polymeric networks of NG1, NG2, NG3 and NG4 is 100, 200, 300 and 400, respectively. This quantity, which will be denoted as *Z*, is also known as the bare charge. Polyelectrolyte chains are linked by 66 tetrafunctional cross-linkers. The cross-linkers of their innermost region are actually linked to four chains. However, the cross-linkers in the outer region only join three or two chains. The number of monomeric units per chain is 30 in the four nanogels. The nanocomposites consist of nanogels and the nanoparticles they absorb. Following the usual notation,^1^ the nanocomposite formed by nanoparticles and certain nanogel will be abbreviated to NP@NG*n* (where *n* is 1, 2, 3 or 4). The electric charge of nanoparticles is −5*e*, which could be representative of slightly charged nanoparticles. The concentration of nanoparticles in the simulation cell is 0.02 mM (the cubic simulation cell with 150 nm sides contains 40 nanoparticles). The total charge of this species is neutralized by monovalent cations (whose concentration is 0.10 mM). In the simulation cell there are also monovalent anions that neutralize the charge of the polymeric network. The simulations were performed on the canonical ensemble. This implies that the number of particles of the different species remains constant.

### Interactions in the CG Model

Excluded volume effects between any pair of beads (monomeric units, cross-linkers, nanoparticles or small ions) are included in the model through the Weeks–Chandler–Andersen (WCA) potential:

1 $$u_{WCA}\left(r\right)=\{\begin{array}{ll} \varepsilon_{WCA}\left({\left(d/r\right)}^{12}+{\left(d/r\right)}^{6}+1/4\right) & r\leq \sqrt[{6}]{2}d\\ 0\; & r>\sqrt[{6}]{2}d \end{array}$$

Here, ε_WCA_ is the parameter characterizing the strength of this interaction, *d* = (*d_i_* + *d_j_* )/2 (where *d_i_* stands for the diameter of species *i*) and *r* is the center-to-center distance between a given pair of particles.

The monomeric units that form the chains (as well as the cross-linkers) are bound by forces derived from the harmonic potential:

2 $$u_{bond}\left(r\right)=0.5\;k_{e}{\left(r-r_{0}\right)}^{2}$$

Here *k*_e_ is the elastic constant of the bond between them and *r*_0_ is the equilibrium distance.

All the charged beads interact through the Coulomb potential:

3 $$u_{elec}\left(r\right)=\frac{Z_{i}Z_{j}e^{2}}{4\pi \varepsilon_{0}\varepsilon_{r}r}$$

Where *Z_i_* is the charge number of species *i*, ε_0_ is the vacuum permittivity, and ε_r_ stands for the relative permittivity of the solvent, whose functional dependence with the absolute temperature (*T*) is given by^38^

4 $$\varepsilon_{r}=\frac{5321}{T}+233.76-0.9297T+0.1417\times 10^{-2}T^{2}-0.8292\times 10^{-6}T^{3}$$

It is important to remark that although this equation includes the effect of the temperature, it does not consider the effect of screening of the charge–charge interactions and image charges. Previous studies have proven that inside dense polymeric systems, low dielectric constant values can be found that may induce a local electrostatic repulsion from image charges of the same sign.^39^

When nonpolar particles are inserted into water, they experience a solvent-mediated attraction (known as hydrophobic force) that tries to aggregate them and increases with temperature.^40^ Since many monomers are nonpolar, the polymers they form collapse when heated. In this work, the hydrophobic force is modeled through an interaction potential^41^ (*u_h_* (*r*)) that consists in a sigmoidal approximation to the square-well potential (previously used by other authors):^42–44^

5 $$u_{h}\left(r\right)=-\frac{\varepsilon_{h}}{2}\left(1-\tanh\left(k_{h}\left(r-r_{h}\right)\right)\right)$$

In this expression, ε*_h_* is the depth of the sigmoidal well, *k_h_* is related to the slope of the sigmoid and *r_h_* is the range of this interaction. The depth of this well increases with temperature according to another sigmoidal function:

6 $$\varepsilon_{h}\left(T\right)=\frac{\varepsilon_{max}}{2}\left(1+\tanh\left(k_{\varepsilon /2}\left(T-T_{\varepsilon /2}\right)\right)\right)$$

Where ε_max_ is the maximum depth of the hydrophobic potential (reached at high temperatures), *T*_ε/2_ is the temperature for which ε*_h_* = ε*_max_*/2 and *k*_ε/2_ proportional to the slope of the function at that point. This potential succeeded in reproducing swelling data of a set of poly­(*N*-isopropylacrylamide)-based microgels.^41^ Consequently, realistic results are expected from it. Other authors have also used different phenomenological solvent-mediated interaction potentials for similar purposes.^8–10,14^

The parameters of the interaction potentials used here are also summarized in Table 1. The values of hydrophobic interaction (*k_h_*, *r_h_*, *T*_ε/2_, *k*_ε/2_ and ε_max_) are identical to those employed to reproduce swelling data of poly­(*N*-isopropylacrylamide)-based microgels^41^ and are shown in Table 1, along with the rest of the parameters that characterize the model interactions. It must also be mentioned that charged monomeric units are usually more hydrophilic than hydrophobic. Hydrophobic forces are therefore expected to be much weaker if a charged monomeric unit is involved. For sake of simplicity, it has been assumed that the hydrophobic interaction is not operative if any of the interacting beads is charged. In other words: the more charged the nanogel is, the fewer hydrophobic groups it has. In any case, it should be kept in mind that the model used here (inspired in temperature-sensitive microgels) is quite simple and quantitative comparisons with experiments (not considered here) could require improvements.

### Moving the Constituent Particles in CG Simulations

Beads were moved applying the Metropolis algorithm. Monomeric units, cross-linkers and small ions (anions and cations) execute single-particle movements; nanoparticles can move on their own or forming a cluster with their nearest counterions. Here, we consider nearest counterions (cations in this case) to those found in a spherical layer around the nanoparticle with a thickness equivalent to twice the diameter of the cation. In any case, it should be emphasized that the cations included in clusters can also move individually (and even leave the cluster), which guarantees the condition of detailed balance. The movement of a set of particles as a rigid body has also been used (alternating with individual moves) in other Monte Carlo simulations to accelerate the sampling of configurations. For example, Dias et al. performed translations of a polymer chain.^45^ The maximum displacement of each bead and cluster was adjusted so that their respective acceptance coefficient approached 50%. During thermalization, expansions and contraction of the whole polymer network and the particles that it contained were also attempted to accelerate this process.

The simulations were performed in a cubic box replicated in the three directions of space using periodic boundary conditions. The length of this box must be long enough to contain the nanogel and the electrical double layer around it. As mentioned previously, the side of the simulation box used here is 150 nm long. According to this, the nanogel/nanocomposite concentration is 2.96 × 10^–7^ nm^–3^. The length of the simulation box is considerably larger than the Debye length for all nanocomposites and for the most highly charged nanogels. The case with the smallest box-length-to-Debye-length ratio corresponds to NG1, which does not reach the value of 3. As will be seen later in a subsequent section, there is reasonably good agreement between certain results obtained for this nanogel from CG simulations (which include periodic images) and a numerical model based on the PB equation (which does not include them). This suggests that the simulation box is large enough even in the most unfavorable case.

Since the simulated system is periodic, its electrostatic energy was calculated by means of Ewald sums following the recommendations proposed by Linse.^46^ In the computation of Ewald sums, strategies for single-particle motions described in another paper were also employed to avoid extremely time-consuming simulations.^47^ These strategies were adapted for cluster movements. Simulations were carried out using a homemade computer code (in C). 3 × 10^8^ and 2 × 10^8^ MC movements were performed for equilibration and statistics, respectively. The evolution of the radius of gyration of the nanogel was monitored to check that an equilibrium value was reached after thermalization.

### Initial Configuration and Thermalization

The initial configuration of the polymer network used in the CG simulations of a given nanogel and its nanocomposite at different temperatures was obtained through a preliminary equilibration process carried out at 20 °C. This process of previous thermalization can be summarized as follows: first, hundreds of cross-linkers were arranged according to the diamond lattice; then a sphere containing the desired number of cross-linkers was drawn, and all those outside it were trimmed off; next, the remaining cross-linkers were joined with straight chains of monomeric units; the necessary counterions were added to neutralize the charge of this network; and, finally, the system was allowed to evolve until it reached a stable size over time. Figure 1a displays the initial configuration of the polymer network corresponding to NG2 (for all temperatures and all its nanocomposites). The initial configuration is completed by placing all the ions and nanoparticles randomly, but avoiding overlap with the polymer network.

**Figure 1:**
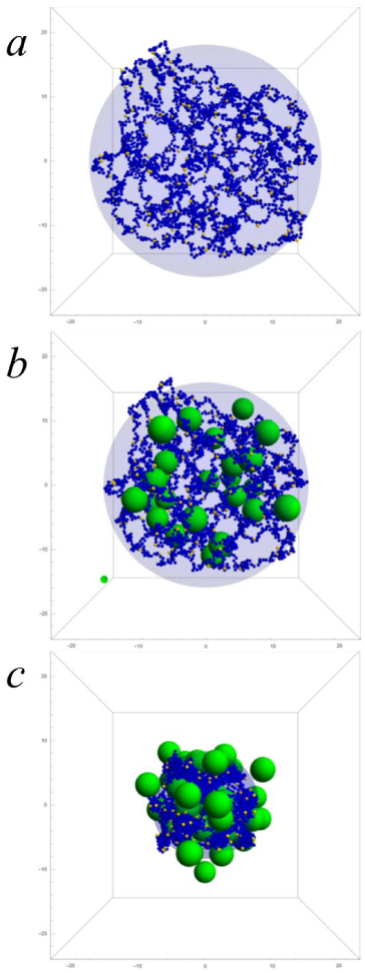
Representative snapshots of the thermalization process of NP@NG2 at 48 °C: (a) initial configuration of the polymer network (obtained from a previous thermalization at 20 °C); (b) configuration after 1.5 × 10^6^ MC steps; (c) configuration at the end of the thermalization process. Blue, yellow and green beads are uncharged monomeric units (and cross-linkers), charged monomeric units and nanoparticles, respectively (small ions are not represented). The transparent blue sphere represents the imaginary border of the polymer network.

The thermalization process includes the absorption of nanoparticles and the collapse of the nanogel, which in turn could cause some desorption. In the early stages of thermalization, the absorption and collapse processes occur simultaneously but at very different rates. This can be seen in Figure 1, which shows the thermalization corresponding to NP@NG2 at 48 °C. After 1.5 × 10^6^ MC steps, the polymeric network is still swollen but it has practically absorbed the nanoparticles that form NP@NG2 (see Figure 1b). This means that the uptake of nanoparticles by the swollen nanogel requires many fewer MC steps than the thermalization process in which the polymer network collapses and expels part of the nanoparticles it contains within. In summary: this thermalization emulates an absorption process when the nanogel is still swollen and the subsequent collapse and desorption process.

### Poisson–Boltzmann Cell Model

The concentrations of ions and other charged species (such as nanoparticles) inside and outside a nanogel can be calculated using the so-called Poisson–Boltzmann cell (PBC) models. Although this idealized representation of reality is much simpler than the CG picture, it sometimes works reasonably well and, in any case, it is instructive to compare its predictions with CG results.

The main hypotheses of the PBC model are the following ones: i) the nanogel is modeled as a sphere (of radius *R*_NG_) whose bare charge is uniformly distributed throughout its volume; ii) ions and nanoparticles can permeate this sphere; iii) the electrostatic interaction experienced by ions and nanoparticles is modeled by a mean field whose potential is obtained by solving a PB equation in a spherical cell:

7 $$\frac{d^{2}\Psi }{dr^{2}}+\frac{2}{r}\frac{d\Psi }{dr}=4\pi l_{B}\left(\frac{3Z\left(1-H\left(r-R_{NG}\right)\right)}{4\pi R_{NG}^{3}}+\underset{i}{\sum }n_{i,0}\exp\left(-z_{i}\Psi \right)\right)$$

Here, Ψ is the dimensionless electrostatic potential, *l*_B_ is the Bjerrum length, *H* is the Heaviside step function, *i* stands for cations, anions and nanoparticles, and *z_i_* and *n_i_*,_0_ are the charge (in elementary units) and the concentration at the border of the spherical cell of species *i*, respectively. The first term inside the parentheses on the right-hand side of eq 7 represents the contribution of the bare charge of the polymer network to the charge density (expressed in elementary units). This term must vanish for *r > R*_NG_; that is why the factor 1 – *H*(*r* – *R*_NG_) is used. The second term inside the parentheses is the contribution of the different ionic species to the charge density (again in elementary units). Eq 7 is solved by assuming that the potential and the electric field are zero at the boundary of the spherical cell.

In its original version, the PB equation considers point ions. However, if the volume of some ionic species (such as nanoparticles) is taken into account, their concentration within the nanogel could not exceed a certain maximum value (*n_i_*,_max_ for species *i*). The modified PB equation that includes this limit on the concentrations of the different ionic species takes the following form:

8 $$\frac{d^{2}\Psi }{dr^{2}}+\frac{2}{r}\frac{d\Psi }{dr}=4\pi l_{B}\left(\frac{3Z\left(1-H\left(r-R_{NG}\right)\right)}{4\pi R_{NG}^{3}}+\frac{\underset{i}{\sum }n_{i,0}\exp\left(-z_{i}\Psi \right)}{1+\underset{i}{\sum }n_{i,0}\exp\left(-z_{i}\Psi \right)/n_{i,max}}\right)$$

*n_i_*,_max_ was estimated as $n_{i,max}\approx \left(1-\varphi_{NG}\right)/d_{i}^{3}$ , where φ_NG_ is the mean polymer volume fraction in the core of the nanogel and *d_i_* the diameter of species *i* (see ref 48 for further details). Eq 8 was numerically solved using the method described by Paunov et al. in the appendix of their work on hydration forces.^49^ It is important to remark that although this model partially includes steric interactions (through *n_i_*,_max_), it ignores spatial correlations between particles.

## Results

### Size of Nanogels

First of all, we will pay attention to the size of the nanogels (in the absence of nanoparticles). Nanogels and nanocomposites are not perfectly spherical particles, but their size can be described in terms of a geometric radius (*R*_NG_) that is calculated from their radius of gyration (*R*_gyr_). The relationship between the two quantities is $R_{NG}=\sqrt{5/3}R_{gyr}$ The radius of gyration is obtained from the positions of the particles (monomeric units and cross-linkers) that make up the polymeric network as

9 $$R_{gyr}^{2}=\left\langle \frac{1}{N}\overset{N}{\underset{i=1}{\sum }}{|{\mathrm{r⃗}}_{i}-{\mathrm{r⃗}}_{CM}|}^{2}\right\rangle$$

Here *N* is the number of particles forming the network, *r⃗_i_* is the vector position of particle *i* and *r⃗*_CM_ is the vector position of the center of mass (CM). The angular brackets denote the average over different conformations.

Figure 2 shows how the geometric radii of (empty) nanogels change with temperature. The error bars were estimated as the standard deviation of three independent runs. This error estimation also applies to the rest of the properties obtained from simulations. As can be seen, the four nanogels simulated here shrink with increasing temperature. Table 2 shows the shrinkage ratio, defined as 1 – *R*_NG_(64 °C)/*R*_NG_(20 °C). As can be concluded, the shrinkage ratio strongly decreases with the bare charge: when the temperature increases from 20 to 64 °C, the radius of NG1 drops by 67% whereas the size of NG4 is only reduced by 37%. Let us remember that the size of a gel results from the balance between the forces that try to swell it and those that induce its collapse. The number of counterions grows with the number of charged groups per chain and the pressure exerted by such counterions contributes to swelling. In addition, we have assumed here that charged beads are not affected by hydrophobic forces, which are responsible for the collapse of gels and nanogels.

**Figure 2:**
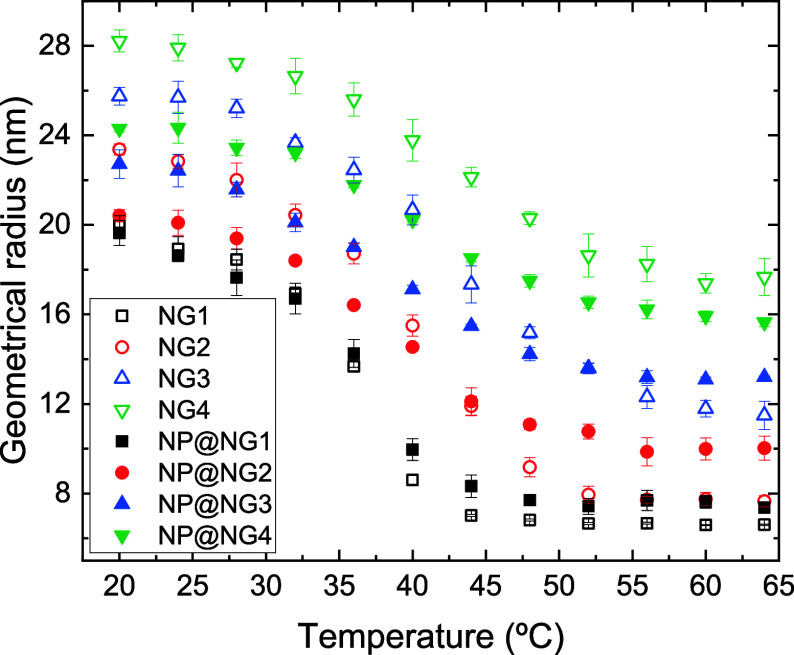
Geometrical radius of nanogels NG1, NG2, NG3 and NG4 (black open squares, red open circles, blue open up triangles and green open down triangles, respectively) and nanocomposites NP@NG1, NP@NG2, NP@NG3 and NP@NG4 (black solid squares, red solid circles, blue solid up triangles and green solid down triangles, respectively) as function of temperature.

**Table 2:** Dependence of Shrinkage Ratio and Desorption Ratio on Bare Charge

Bare charge	Shrinkage ratio of nanogels	Shrinkage ratio of nanocomposites	Desorption ratio
100	0.67	0.62	0.88
200	0.67	0.58	0.52
300	0.55	0.42	0.15
400	0.37	0.36	0.05

### Electrostatic Potential of Nanogels

As mentioned above, both the nanogel and the nanoparticles have electrical charge. To get an idea of the electrostatic forces between them, the spherically averaged electrostatic potential (*ψ*) was computed as

10 $$\psi \left(r\right)=-\int_{L/2}^{r}E\left(r\right)dr$$

In this expression, *r* is the distance to the CM, *L* is the length of the simulation cell, *E*(*r*) is the spherically averaged electric field, which in turn can be obtained from the net charge enclosed by a sphere of radius *r* by applying Gauss’ law.

Figure 3 shows the dimensionless electrostatic potential as a function of the distance to the CM at 24 °C for the four nanogels. The dimensionless potential is defined as *e*ψ/*k*_B_ *T* (where *k*_B_ is Boltzmann’s constant). This potential is zero at the border of the simulation cell but increases rapidly as we approach the nanogel. Therefore, an oppositely charged nanoparticle in the proximity of the nanogel would be drawn into it. Obviously, these attractive electrostatic forces can be responsible for nanoparticle absorption. In any case, recent simulations and experiments have proven that nanoparticles can enter like charged nanogels by diffusion if the nanoparticle concentration is high enough.^50^ It should also be noted that the electrostatic potential tends to a constant value in the core of the nanogel (approximately, *r*/*R*_NG_ < 0.3). Consequently, electrostatic forces are negligible in this region. Figure 3 also includes the electrostatic potential at 56 °C. As can be seen, this physical magnitude increases considerably with temperature, particularly for NG1 and NG2, the two nanogels whose relative shrinkage is greater.

**Figure 3:**
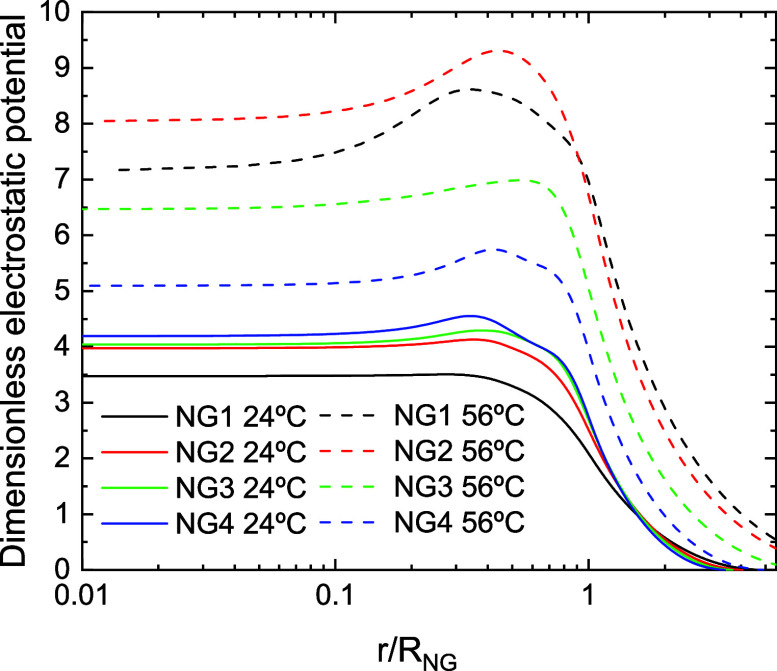
Dimensionless electrostatic potential as a function of the normalized distance from the CM for NG1, NG2, NG3 and NG4 at 24 °C (black, red, blue and green solid line, respectively) and NG1, NG2, NG3 and NG4 at 56 °C (black, red, blue and green dashed line, respectively).

### Nanoparticles in Nanocomposites

As mentioned above, one of the main objectives of this work is to find out to what extent thermo-shrinking nanogels can expel nanoparticles housed in them when the temperature is increased. Figure 4 displays the number of nanoparticles that remains inside the polymer network at different temperatures. We consider that a nanoparticle is located inside a nanogel if the distance between their respective centers of mass is less than the geometric radius of the polymer network. Figure 4 also suggests that the degree of desorption of nanoparticles after collapse of the nanocomposites depends on their bare charge. To prove this quantitatively, let us define the desorption ratio as the quotient between the number of particles expelled (at 64 °C) and the number of particles absorbed (at 20 °C). This ratio is also included in Table 2. As can be inferred from this table, the desorption rate also decreases with the bare charge. As the temperature increases, NP@NG1 expels about 88% of the nanoparticles it contains in its swollen state. At the other extreme, NP@NG4 only ejects 5%.

**Figure 4:**
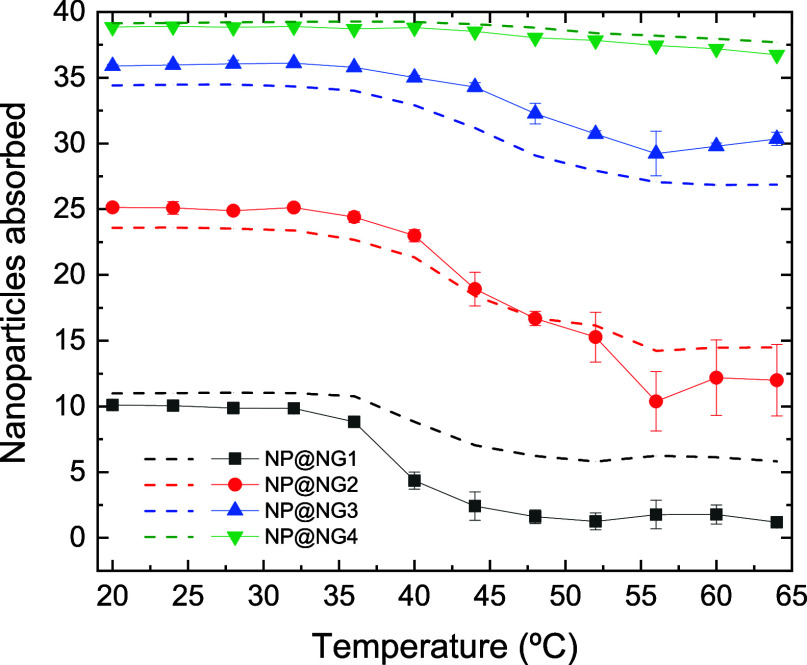
Number of nanoparticles that remain inside the polymer network for nanocomposites NP@NG1, NP@NG2, NP@NG3 and NP@NG4 (black solid squares, red solid circles, blue solid up triangles and green solid down triangles, respectively) as a function of temperature. Dashed lines stand for the predictions of a Poisson–Boltzmann cell model (see text and ref 48 for further details).

At this point, the following question arises: what interaction allows NG1 to expel almost all the nanoparticles? According to our model, there are only two types of direct interactions between nanoparticles and the constituents of the polymeric network: steric and electrostatic. We should also keep in mind that the electrostatic forces between the nanoparticles and the polyelectrolyte chains are attractive because they are oppositely charged. This therefore suggests that steric forces are responsible for the expulsion of nanoparticles when the polymer network collapses. This hypothesis can be supported by comparing with situations in which steric forces are much weaker. Ahualli et al. simulated the absorption of monovalent and trivalent small ions into a negatively charged nanogel whose thermal response in size is quite similar to that of NG1.^48^ These researchers found that approximately one-third of the monovalent ions and half of the trivalent ions remained inside the nanogel after collapse. Since our nanoparticles are larger than those ions, we should conclude that steric forces are responsible for the desorption observed for NP@NG1.

It is interesting to compare the number of absorbed particles obtained by CG simulations with the PBC predictions. Eq 8 was solved assuming that the volume of the spherical cell of the PBC model is equal to that of the CG simulation box. As can be seen, the PBC model predicts the number of absorbed nanoparticles reasonably well (despite its simplicity) for NP@NG2, NP@NG3 and NP@NG4. Since steric correlations are ignored, this means that absorption is largely driven by electrostatic forces. In the case of NP@NG1, the model only works at low temperatures. This suggests that steric correlations should not be ignored at high temperatures.

With regard to Figure 4, it is also important to remember that the number of nanogels/nanocomposites per unit of volume is fixed in these simulations and the nanogel/nanocomposite concentration is 2.96 × 10^–7^ nm^–3^. The number of absorbed nanoparticles may depend on this concentration, particularly in the case of nanogels with high absorption capacity, such as NG4.

### Size of Nanocomposites

Before going into detail about the mechanism behind this expulsion, it is interesting to analyze whether the presence of nanoparticles inside the nanogels modifies their size. Figure 2 also includes geometric radii of the four nanocomposites at different temperatures. As can be seen, at low temperatures the size of the nanocomposite is generally smaller than that of the corresponding nanogel. To justify this behavior, it should be taken into account that when negatively charged nanoparticles enter the nanogel, a considerable number of monovalent anions are expelled from it. Therefore, the total number of charged species (nanoparticles and ions) within the network decreases and the osmotic pressure they exert also decreases. This would cause the nanocomposite to deswell a little. At high temperatures, if the number of nanoparticles remaining inside the polymeric network is large enough, such nanoparticles could prevent the nanocomposite from shrinking to the size of the empty nanogel. This is clearly seen in the case of the NP@NG2 nanocomposite and, to a lesser extent, in the NP@NG1 and NP@NG3 nanocomposites.

### Nanoparticle Distribution

The spatial distribution of the nanoparticles within the nanogels also deserves some attention. Figure 5 shows the spherically averaged concentration (or number density) of nanoparticles in the nanocomposite NP@NG2 (as a representative example) at 36, 40, 44, and 52 °C as a function of the distance to its CM normalized by the radius of the polymer network of such nanocomposite. According to Figure 4, the temperature at which these nanoparticles begin to be expelled from NP@NG2 is 36 °C. As can be concluded from Figure 5, at this temperature nanoparticles are concentrated preferentially in the central part of NG2. It should be mentioned, however, that nanoparticles with greater charges can be structured in shells at different distances from the CM.^2^ Figure 5 also shows that the tendency of nanoparticles to accumulate in the center of the nanogel becomes more pronounced when the temperature rises to 40 °C. It should be stressed, however, that nanoparticles begin to structure into layers if the temperature continues to rise. For example, the distribution function at 44 °C exhibits two peaks in the middle of the nanogel. The first of these, which is the highest, is not right in the center. A peak also appears near the surface. This means that the particles expelled from the interior tend to stay near the imaginary boundary of the polymeric network due to attractive electrostatic forces. At higher temperatures (52 and 56 °C), the first peak moves slightly further from the center. Furthermore, it should be emphasized that the concentration of nanoparticles near the surface increases considerably because the expelled nanoparticles are retained in the vicinity of the polymer network by the attractive electrostatic interaction.

**Figure 5:**
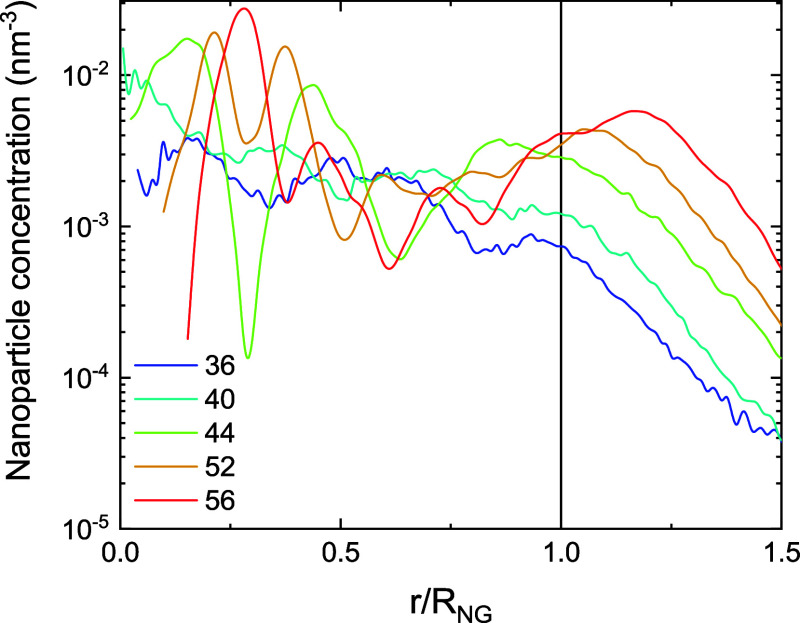
Spherically averaged concentrations of nanoparticles as a function of the normalized distance from the center of mass for NP@NG2 at 36, 40, 44, 52, and 56 °C (blue, cyan, light green, sandy brown, and red line, respectively).

As mentioned above, Figure 5 reveals that the nanoparticles absorbed inside a nanogel appear to be structured into layers when the polymeric network collapses. The nanoparticle–nanoparticle radial distribution function (*g*_NP–NP_(*r*)) provides additional information about this spatial order. Figure 6 displays (*g*_NP–NP_(*r*) for NP@NG2 at 36, 40, 44, 52, and 56 °C. As can be clearly seen, this function shows well-defined peaks at 5 nm for 44, 52, and 56 °C. Such peaks prove that the nanoparticles absorbed into the collapsing nanogel are very close to each other. It should also be noted that the height of these peaks (related to the degree of spatial order) grows with temperature. In fact, at 56 °C a second (less defined) peak is even observed at about 10 nm. The close packing of nanoparticles that gives rise to these peaks is also shown in the inner snapshot. It should be noted that the high degree of spatial correlation reported for collapsing nanocomposites is usually neglected in mean-field approaches, such as the PBC.

**Figure 6:**
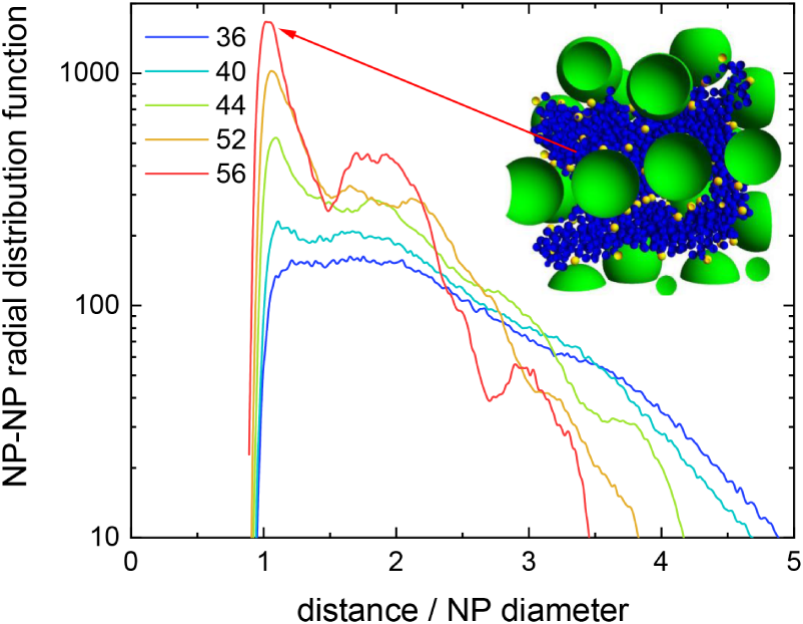
Nanoparticle–nanoparticle radial distribution function as a function of the distance from the center of mass (normalized by the NP diameter) for NP@NG2 at 36, 40, 44, 52, and 56 °C (blue, cyan, light green, sandy brown, and red line, respectively). Inset: cross sections of NP@NG2 at 56 °C. Blue, yellow, and green beads represent uncharged monomeric units (or cross-linkers), charged monomeric units and nanoparticles, respectively. The frontal plane passes through the center of the polymer network.

At this point it is important to mention that, during the collapse process, the nanogel goes through highly heterogeneous states, which were extensively studied (by using coarse-grained simulations) for hydrogels in poor solvents by Mann et al.^14^ The inset of Figure 6 is a cross section of NP@NG2 at 56 °C showing such heterogeneities in the presence of nanoparticles. As can be seen, the monomeric units form compact globules that coexist with voids. This inset also displays how these nanoparticles leak through the voids and even break through by deforming the partially collapsed polymer.

In relation to the strong correlations that appear between the nanoparticles when NP@NG2 collapses, it is worth going back for a moment to Figure 4 and observing the large error bars that the number of nanoparticles contained in this nanocomposite has at high temperatures. These fluctuations could be related to the influence that strong correlations between nanoparticles would have in the final phase of the collapse process (which takes place during thermalization). The effects of these correlations would be more noticeable in systems with a high degree of shrinkage that also retain a considerable number of nanoparticles (such as NP@NG2).

### Net Charge and Surface Electrostatic Potential

It is interesting to analyze how some electrical properties of nanogel and nanocomposites (i.e., net charge and surface potential) change upon heating. Let us first analyze the behavior of nanogels. Figure 7 shows the spherically averaged net charge (expressed as number of elementary charges, *Z*_net_) as a function of temperature for the four nanogels. *Z*_net_ is computed as the total charge enclosed by the imaginary surface of the nanogel or nanocomposite (the sphere of radius *R*_NG_). As can be seen, this property decreases with temperature for the empty nanogels, which can be explained by the absorption of counterions during the collapse process. Other researchers have measured the effective charge of microgels at low ionic strength using different techniques and also conclude that this charge decreases with temperature,^51,52^ in clear agreement with our simulation results.

**Figure 7:**
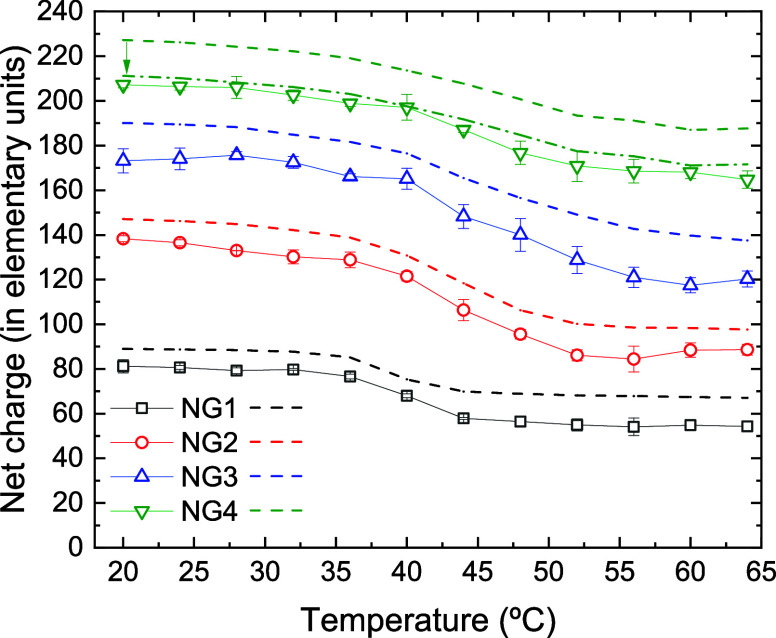
Spherically averaged net charge (expressed as number of elementary charges) obtained from simulations for nanogels NG1, NG2, NG3 and NG4 (black open squares, red open circles, blue open up triangles and green open down triangles, respectively) as a function of temperature. The figure also includes the predictions of the PBC model for the same nanogels (black, red, blue, and green dashed lines, respectively). The green dot-dashed line represents the PBC prediction for NG4 after subtracting the external bare charge and the arrow symbolizes such correction (see text for further details).

Figure 7 also includes the net charge predictions obtained from the PBC model. As can be seen, this model overestimates the charge value obtained from CG simulation. This overestimation can largely be attributed to the fact that the model assumes that the entire bare charge of the nanogel is contained in the imaginary sphere of radius *R*_NG_. However, in simulated nanogels there is always a fraction of charged groups outside that sphere. For example, in the case of the NG4 nanogel at 20 °C, there are about 16 charged groups (on average) that are outside that sphere. When calculating the net charge, the CG simulation code does not account for these groups. However, the PBC model assumes that these groups are within the sphere of radius *R*_NG_, so it includes them in the calculation of the net charge. To compare on equal terms, the electrical charge of these groups should be subtracted from the PBC model prediction. Quantitative agreement with the CG simulation would improve considerably in that case (as can be seen in the Figure 7 for NG4).

The dimensionless spherically averaged surface electrostatic potential (*e*ψ*(R*_NG_ *)/k*_B_ *T*) of the four nanogels is plotted as a function of temperature in Figure 8. As can be seen, the electrostatic surface potential increases in all cases when nanogels collapse. The less charge the polymer network has, the greater this increase. This is probably because the electrostatic surface potential is very sensitive to the size of the nanogel and this property undergoes larger changes when the bare charge of the nanogel decreases. Figure 8 also includes the predictions of the PBC model for the surface electrostatic potential. The agreement with CG simulation results is reasonably good, except in the case of NG1 at high temperatures.

**Figure 8:**
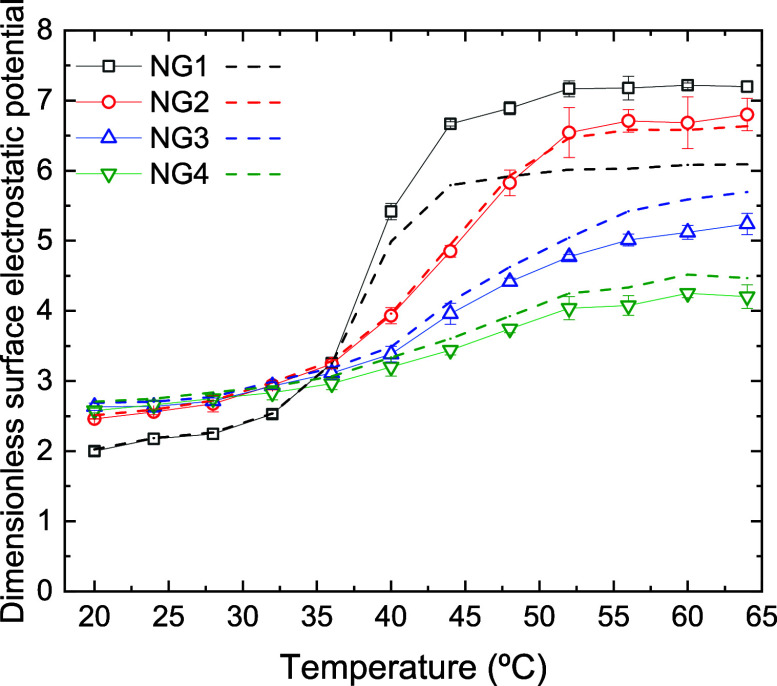
Dimensionless spherically averaged surface electrostatic potential of NG1, NG2, NG3 and NG4 (black open squares, red open circles, blue open up triangles, and green open down triangles, respectively) as a function of temperature. The figure also includes the predictions of the PBC model for the same nanocomposites (black, red, blue, and green dashed lines, respectively).

Once the electrical properties of nanogels have been analyzed, let us examine what happens in the case of the corresponding nanocomposites. Figure 9 shows that the net charge of nanocomposites behaves very differently from that of nanogels: *Z*_net_ increases with temperature (instead of decreasing) for NP@NG1, NP@NG2 and NP@NG3 and hardly varies for NP@NG4. Additional simulation data (not explicitly shown here) reveal that (i) the increase in the net charge of NP@NG1 and NP@NG2 when these nanocomposites collapse can be justified (to a great extent) by the expulsion of nanoparticles, which is not counteracted by the absorption of counterions; (ii) in the case of NP@NG3, the expulsion of nanoparticles is partially counteracted by the absorption of counterions; (iii) in the case of NP@NG4, more counterions enter than required to neutralize the charge of the expelled nanoparticles. This figure also includes the PBC model predictions for the net charge of the nanocomposites. From a qualitative point of view, it should be highlighted that the PBC model correctly predicts that the net charge of NP@NG1, NP@NG2 and NP@NG3 should increase when these systems collapse. However, it fails to predict the behavior of NP@NG4. Quantitatively speaking, the PBC model predicts the net charge of NP@NG1 very well, but its predictions worsen as the bare charge of the nanogels increases. Perhaps these quantitative differences could be reduced if the electric charge of the groups that are outside the sphere of radius *R*_NG_ is subtracted from the net charge of the PBC model. To find out how far this is true, the correction of external bare charge was also applied to NP@NG4. Such a correction improves the model prediction at low temperatures, but the improvement is insufficient at high temperatures.

**Figure 9:**
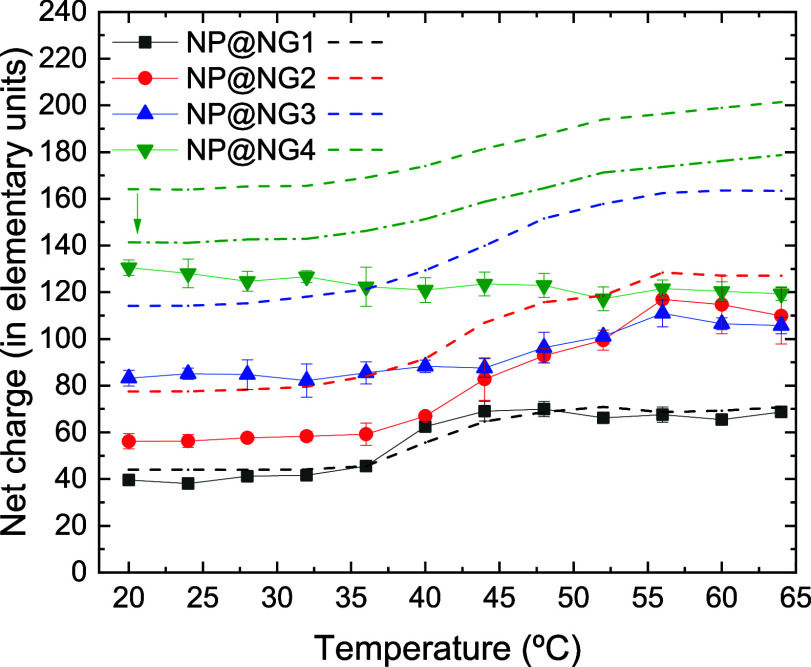
Spherically averaged net charge (expressed as number of elementary charges) obtained from simulations for nanocomposites NP@NG1, NP@NG2, NP@NG3, and NP@NG4 (black solid squares, red solid circles, blue solid up triangles, and green solid down triangles, respectively) as a function of temperature. The figure also includes the predictions of the PBC model for the same nanocomposites (black, red, blue, and green dashed lines, respectively). The green dot-dashed line represents the PBC prediction for NG4 after subtracting the external bare charge and the arrow symbolizes such correction (see text for further details).

In relation to this quantitative and qualitative disagreement between the PBC theory and the CG simulation results, it should be kept in mind that the PBC model involves much greater simplifications than the CG model. For example, it does not consider the internal structure of the nanogel. What is more, the PBC model assumes that the distributions of monomers, ions, and nanoparticles only depend on *r*, the distance to the center of the nanogel. In other words, spherical symmetry is assumed. Accordingly, this model is expected to fail with particles of large charge and/or size, such as nanoparticles. Strong interactions between them can lead to a spatial arrangement that does not have spherical symmetry. An illustrative example of the absence of spherical symmetry is shown in the snapshot in Figure 6. Accordingly, we can conclude that the PBC model fails for the case of nanocomposites because strong correlations due to nanoparticles are ignored in directions perpendicular to the radial one.

With regard to Figures 7 and 9, it is instructive to find out to what extent small counterions and nanoparticles can neutralize the bare charge of nanogels and nanocomposites. This can be done by plotting the net charge normalized by the bare charge. Figure 10 shows this ratio as a function of temperature. When the polymer network is swollen (at low temperatures), it is clear that the ability to neutralize the bare charge is greater for nanocomposites, since their ratio *Z*_net_/*Z* is lower than that of nanogels. This is because nanoparticles have higher charges than small monovalent ions. In fact, nanoparticles with charges greater than that used in this study can completely neutralize the charge of the polymer network and even reverse the sign of the net charge.^28^ This phenomenon, known as overcharging, charge inversion or charge reversal, is not observed here because nanoparticles are not charged enough. At high temperatures, however, there is no clear pattern that allows us to say that nanocomposites neutralize the bare charge more efficiently.

**Figure 10:**
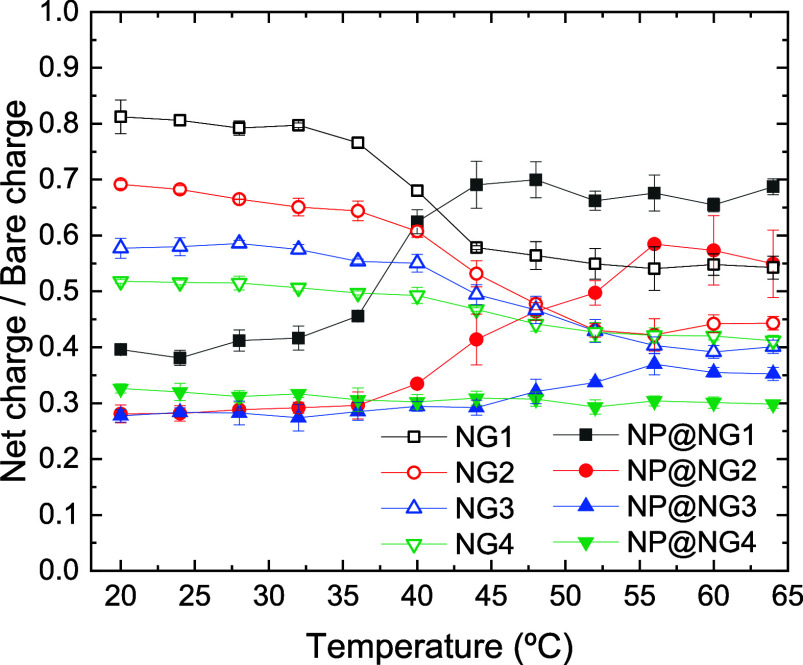
Net-charge-to-bare-charge ratio of nanogels NG1, NG2, NG3 and NG4 (black open squares, red open circles, blue open up triangles, and green open down triangles, respectively) and nanocomposites NP@NG1, NP@NG2, NP@NG3 and NP@NG4 (black solid squares, red solid circles, blue solid up triangles, and green solid down triangles, respectively) as a function of temperature.

Figure 11 displays the dimensionless surface electrostatic potential of the four nanocomposites as a function of temperature. As can be inferred, this quantity also increases in all cases when the nanocomposites collapse upon heating. However, the curves are not ordered with the bare charge of the polymer network. Their behavior is much more difficult to describe and justify. Furthermore, the predictions of the PBC model (also plotted in the figure) deviate considerably from the simulation results, mostly at high temperatures. In relation to these discrepancies, it should be stressed again that when nanocomposites collapse, the nanoparticles contained in them might experience strong steric and electrostatic correlations, which are partly ignored by the PBC model. The steric correlations between the nanoparticles and the polymeric network are also ignored. Furthermore, the electrostatic potential varies rapidly in the vicinity of the nanogel surface, so the effects of these omissions in the model could be considerably intensified.

**Figure 11:**
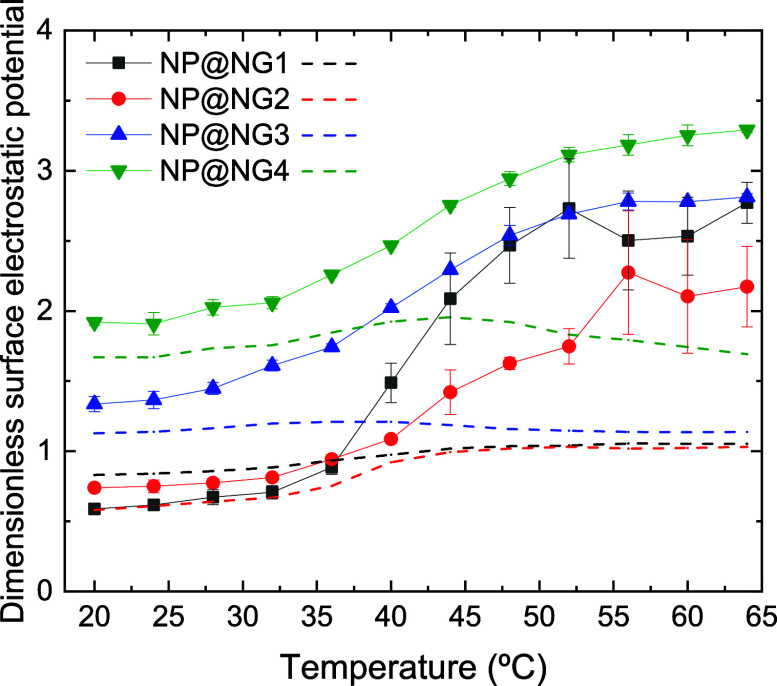
Dimensionless spherically averaged surface electrostatic potential of NP@NG1, NP@NG2, NP@NG3, and NP@NG4 (black solid squares, red solid circles, blue solid up triangles, and green solid down triangles, respectively) as a function of temperature. The figure also includes the predictions of the PBC model for the same nanocomposites (black, red, blue, and green dashed lines, respectively).

### Additional Considerations

Before presenting the conclusions, it would be interesting to outline some final considerations that could be useful in future work. The first of these concerns the definition of the radius of a nanogel. These polymeric networks are not perfectly spherical and, therefore, defining a radius for them is not a trivial matter. In this study, the radius was calculated using eq 9 (proposed by Claudio et al.^53^), which is based on the radius of gyration of a solid sphere. The great advantage of this definition is that the radius of gyration of polymers can be determined experimentally. However, our results have revealed that this definition might not work well when dealing with electrical properties, such as net charge or surface electrostatic potential. For example, charged monomers have been found to be present at distances greater than the radius given by eq 9. This is not surprising, since this definition of radius is based solely on mass distribution but completely ignores that electrical properties undergo abrupt changes at the boundary of the polymer network. The electrostatic potential and the distribution of charged monomers drop rapidly in that region. In the case of collapsed nanocomposites, there may also be a high concentration of nanoparticles. These changes could be used to formulate alternative definitions of the radius of nanogels (and nanocomposites). For example, Figure 3 shows that the electrostatic potential of the nanogels exhibits an inflection point near their surface. It would be worthwhile to investigate whether the radius associated with this point provides more consistent results for the electrical properties. In any case, it would also be desirable for the new definition of radius to be experimentally accessible.

The second consideration relates to the effect of boundary conditions on nanoparticle desorption. As mentioned previously, our GC simulations were performed on the canonical ensemble. Therefore, the number of nanoparticles in the simulation box remains constant. In the case of moderately or highly collapsed nanogels, many of the nanoparticles accumulate near the surface of the polymer network (see Figure 1c, Figure 5 and inset of Figure 6). Such accumulation could prevent (at least partially) the release of nanoparticles inside the nanocomposites. This raises the question of whether removing the external nanoparticles from the simulation box enhances the desorption of the internal ones. To find out if this effect is quantitatively relevant, a preliminary test has been performed with NP@NG2 and NP@NG4, at 64 °C. In both cases, the simulations have been extended (from their final configuration) including an algorithm that removes nanoparticles that are outside the nanocomposite. When the number of particles in the simulation box is halved, the number of nanoparticles within the polymer network falls to 36% and 25% (rounded) for NP@NG2 and NP@NG4, respectively. This preliminary test suggests that the effect of boundary conditions (emulating different experimental conditions) should be studied in depth.

## Conclusions

In this work, a coarse-grained model has been used to perform MC simulations of thermosensitive nanocomposites. Only four basic interactions are considered in this CG picture: steric, electrostatic, hydrophobic and monomer–monomer bonding interactions. Despite the simplicity of this model, the nanocomposites simulated here exhibit a rich behavior when varying the electrical charge of their polymeric network. On the one hand, the nanocomposites with low bare charge undergo significant size reductions when temperature increases and are able to expel many of the nanoparticles they have absorbed by electrostatic attraction. The pronounced decrease in size also leads to a considerable increase in the surface electrostatic potential. On the other hand, the nanocomposites with high bare charge vary less in size and, therefore, have a lower capacity to expel the nanoparticles they contain when heated.

Regarding the net charge, simulations show that the thermal behavior of nanocomposites differs from that of nanogels. The net charge of the nanogels decreases when they collapse. In contrast, the net charge of less charged nanocomposites tends to increase as they shrink. The behavior of the surface potential is more complex and cannot be ordered according to the bare charge because it depends on both the net charge and the size of the nanocomposite.

A mean-field theory (based on the PB equation) has also been employed in our study to predict the number of absorbed nanoparticles, the net charge and the electrostatic surface potential of nanocomposites. The PBC theory ignores the internal structure of the polymeric network as well as steric and electrostatic correlations between charged species, so it is not computationally expensive. This very simple model works reasonably well with (empty) nanogels, but it may fail when applied to nanocomposites, particularly when they collapse and steric and electrostatic interactions induce strong correlations between nanoparticles.

The only interaction with certain specificity is the hydrophobic one, whose parameters reproduce swelling data of poly­(*N*-isopropylacrylamide). The remaining interactions are nonspecific, i.e., they do not depend on the chemical nature of the nanogel or nanoparticles. Therefore, the conclusions reached here are potentially applicable to a wide variety of nanocomposites (as long as other specific interactions are negligible).
